# Diagnostic Challenges in HHV-8-Associated Multicentric Castleman Disease in a Patient with Prior Kaposi Sarcoma

**DOI:** 10.3390/dermatopathology12040033

**Published:** 2025-10-02

**Authors:** Seraphima S. Sidhom, Luke A. Laconi, Christopher A. LaFond, Steven C. Weindorf

**Affiliations:** 1 Michigan State University College of Human Medicine, Grand Rapids, MI 49503, USA; laconilu@msu.edu (L.A.L.);; 2 Department of General Surgery, Munson Medical Center, Traverse City, MI 49684, USA; 3 Department of Pathology, Munson Medical Center, Traverse City, MI 49684, USA; 4 Grand Traverse Pathology, PLLC, Traverse City, MI 49684, USA

**Keywords:** multicentric Castleman disease, HHV-8, Kaposi sarcoma, HIV

## Abstract

Human herpesvirus-8 (HHV-8)-associated multicentric Castleman disease (MCD) is a rare lymphoproliferative disorder with systemic and cutaneous manifestations that can be diagnostically challenging, especially in immunocompromised patients. We report the case of a 68-year-old man with HIV and biopsy-proven Kaposi sarcoma (KS), who developed progressive fevers, night sweats, weight loss, and fatigue, accompanied by diffuse lymphadenopathy, splenomegaly, and new erythematous and hyperpigmented lesions shortly after intravenous immunoglobulin therapy for Guillain–Barré syndrome. A laboratory evaluation revealed that the patient had elevated total protein and polyclonal hypergammaglobulinemia, without monoclonality. Imaging demonstrated widespread lymphadenopathy and splenomegaly. A core lymph node biopsy showed polytypic plasmacytosis, but was non-diagnostic. Given the ongoing symptoms, an excisional biopsy was performed, revealing regressed germinal centers with increased interfollicular vascularity, mantle zone “onion skinning,” and HHV-8 LANA-1 nuclear positivity, establishing the diagnosis of HHV-8-associated MCD. Rituximab monotherapy was initiated, resulting in clinical improvement, resolution of the constitutional symptoms, and stabilization of ascites. This case highlights the importance of maintaining a high index of suspicion for MCD in patients with KS who develop new systemic or cutaneous findings, the limitations of a core biopsy, and the value of a timely excisional biopsy in guiding diagnosis and treatment.

## 1. Introduction

Kaposi sarcoma-associated herpesvirus (KSHV), or human herpesvirus-8 (HHV-8), is a gamma-herpes virus that plays a major role in the development of various lymphoproliferative and neoplastic disorders. It has been frequently linked to Kaposi sarcoma (KS), primary effusion lymphoma (PEL), and multicentric Castleman disease (MCD). The virus is spread through bodily fluids, such as saliva and sexual contact. Once acquired, HHV-8 establishes a lifelong latent infection within B lymphocytes and plasmablasts. After infection, HHV-8 creates a latent infection in B cells and plasmablasts that lasts a lifetime. The latent-associated nuclear antigen-1 (LANA-1) gene is one of the few genes that the virus expresses during latency. It is essential for maintaining the viral episome and avoiding host immune responses. Immune compromise frequently causes reactivation into the lytic cycle, especially in people with Human Immunodeficiency Virus/Acquired Immunodeficiency Syndrome (HIV/AIDS) [1,2].

The viral protein vIL-6 in HHV-8-associated MCD functions similarly to human IL-6, causing a strong and dysregulated inflammatory response that results in polyclonal plasmacytosis, B cell proliferation, and the disease’s distinctive clinical manifestations. This is very different from idiopathic MCD, which is believed to be caused by human IL-6 and is not linked to HHV-8. Non-specific systemic inflammatory symptoms, like polyclonal hypergammaglobulinemia, splenomegaly, ascites, and generalized lymphadenopathy, are common in MCD patients [3].

The reactivation of HHV-8 can cause aggressive disease manifestations that resemble other systemic illnesses in patients with weakened immune systems, especially those with advanced HIV or a history of oncologic disease. These patients may present with vague symptoms, such as fever, weight loss, or fatigue, delaying a definitive diagnosis. Furthermore, clinical interpretation may be complicated by overlapping features with other HHV-8-associated pathologies, such as PEL or Kaposi sarcoma. Therefore, when faced with unexplained systemic inflammation, especially in high-risk populations, clinicians must remain on the lookout for HHV-8-associated conditions when making differential diagnoses.

Diagnosing MCD can be quite challenging, as the clinical and laboratory features often overlap with various malignancies, autoimmune diseases, and infections [1]. Due to the rarity of the disease and its non-specific presentation, HHV-8-associated MCD can frequently be overlooked, especially in patients with complex immune dysregulation or prior viral-related malignancies [4]. Early treatment with anti-CD20 therapy, such as rituximab, can significantly improve outcomes [5]. However, given that the estimated 5-year overall survival rate is approximately 65%, timely detection and accurate diagnosis are critical [6]. Although awareness is increasing among dermatologists, pathologists, and oncologists, diagnostic strategies for HHV-8-associated MCD can still be improved [6].

This case illustrates the clinical presentation, diagnostic process, and treatment response of HHV-8-associated MCD in a patient, following treatment for Guillain–Barré syndrome. It highlights the importance of maintaining a high index of suspicion for this rare but aggressive disease, especially in patients with predisposing factors, such as HIV infection and a history of Kaposi sarcoma, and underscores the critical role of a timely and appropriate biopsy in achieving a definitive diagnosis.

## 2. Case Report

We report the case of a 68-year-old man with a known history of HIV infection and biopsy-confirmed Kaposi sarcoma (KS). He initially presented with rapidly appearing violaceous, purple lesions on the nose, chest, and back. HIV testing at that time was positive, and a subsequent skin biopsy confirmed KS. The patient was started on antiretroviral therapy, namely with bictegravir/emtricitabine/tenofovir alafenamide (Biktarvy), and remained clinically stable for nearly two years. He then re-presented with progressive constitutional symptoms, including fevers, night sweats, unintentional weight loss, and worsening fatigue. On examination, he was found to have diffuse cervical lymphadenopathy and splenomegaly, in addition to new cutaneous findings of scattered erythematous lesions on the fingers, abdomen, and posterior trunk, along with a brown hyperpigmented lesion in the axilla. These concerning features prompted further evaluation.

He had recently completed intravenous immunoglobulin (IVIG) therapy for Guillain–Barré syndrome (GBS), a rare neurological disorder, characterized by the acute onset of muscle weakness, due to immune-mediated damage to peripheral nerves. The IVIG therapy, while crucial for managing GBS, could have transiently influenced his immune system, potentially masking or altering the presentation of other underlying conditions. This recent GBS diagnosis and its treatment added a layer of complexity to the diagnostic process, as it necessitated distinguishing between symptoms related to his neurological recovery and those indicative of a new, systemic issue. His ongoing fatigue and weight loss, despite GBS treatment, strongly suggested an alternative or co-occurring pathology. On examination, he was noted to have splenomegaly and cervical lymphadenopathy. These findings, along with his constitutional symptoms, raised immediate concern about an underlying systemic process.

The laboratory workup revealed that the patient had elevated total protein, polyclonal hypergammaglobulinemia, and a preserved CD4 count. The polyclonal hypergammaglobulinemia, specifically, pointed towards a broad, non-clonal activation of B lymphocytes, a common finding in inflammatory or infectious conditions, but also a hallmark of Castleman disease. Serum protein electrophoresis and immunofixation showed increased IgG and both kappa and lambda light chains, without evidence of monoclonality, further supporting the polyclonal nature of the hypergammaglobulinemia and effectively ruling out multiple myeloma and other plasma cell dyscrasias. A bone marrow biopsy demonstrated mild polytypic plasmacytosis without malignant infiltration, indicating an increase in diverse plasma cells, again consistent with a reactive or inflammatory process rather than a true malignancy of plasma cells. A CT scan of the chest, abdomen, and pelvis showed widespread lymphadenopathy and splenomegaly. A core biopsy of a cervical lymph node revealed polytypic plasmacytosis, with features suggestive of Castleman disease.

**Figure 1:**
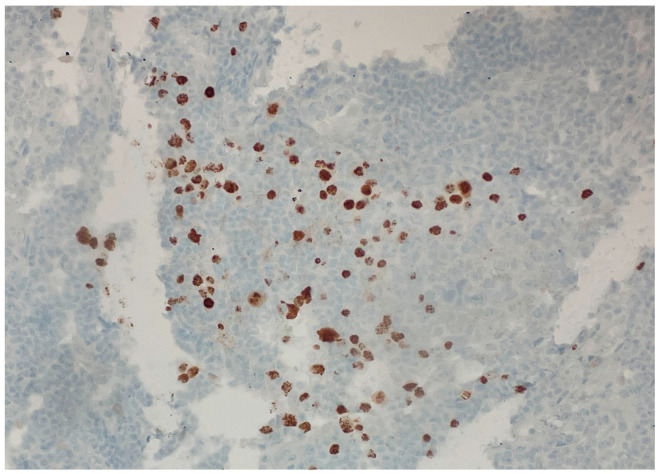
**Immunohistochemical staining demonstrating HHV-8 (LANA-1) positivity. (40×).** Scattered brown nuclear staining in lymphoid cells indicates the presence of latent HHV-8 infection, consistent with HHV-8-associated multicentric Castleman disease. This staining pattern confirms the presence of latent nuclear antigen-1 (LANA-1), a specific marker for HHV-8-infected cells.

**Figure 2:**
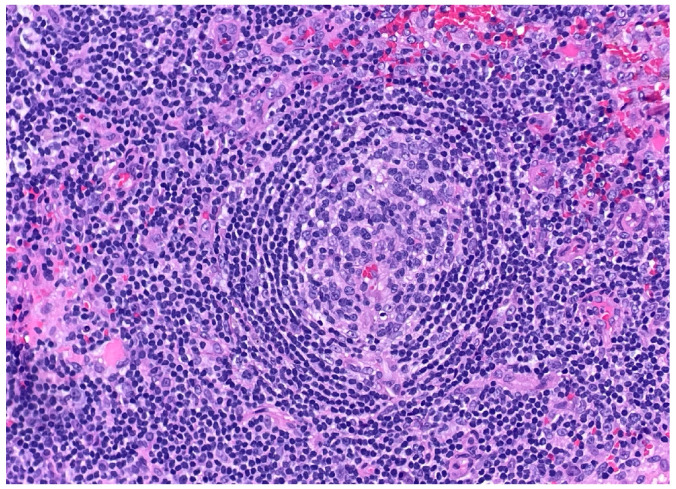
**Higher-power view of secondary follicle showing characteristic “onion skinning” of mantle zone lymphocytes. (H&E, 20×).** This histopathologic feature can be seen in all types of Castleman disease. The concentric layering of small lymphocytes around atrophic germinal centers resembles an “onion skin” pattern.

**Figure 3:**
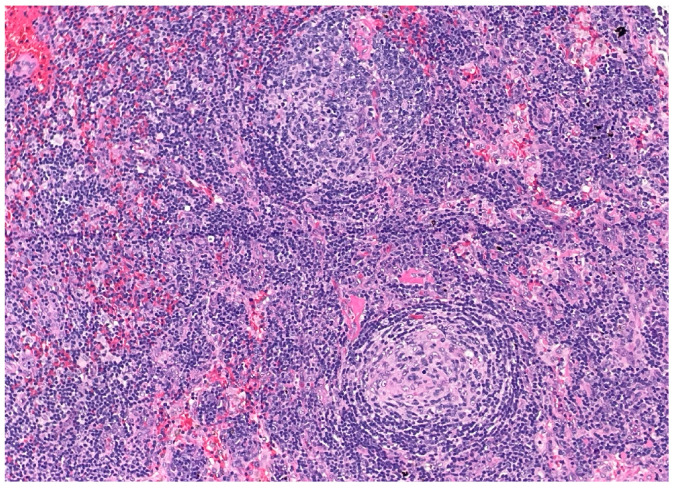
**Low-power view of secondary follicles showing regressed germinal center and prominent interfollicular vascularity. (H&E, 10×).** The follicle on the bottom shows an atrophic and partially hyalinized germinal center. The follicle on the top shows a vessel penetrating the germinal center (“lollipop” feature). These features can be seen in all types of Castleman disease.

Over the following weeks, the patient developed ascites and worsening constitutional symptoms. An excisional biopsy of a lymph node was subsequently performed and demonstrated HHV-8-positive MCD, confirmed by immunohistochemical staining for LANA-1, showing scattered nuclear positivity in lymphoid cells (Figure 1). The histology also revealed characteristic “onion skinning” of mantle zone lymphocytes (Figure 2) and regressed germinal centers with increased interfollicular vascularity and hyalinization (Figure 3), findings consistent with HHV-8-associated MCD. Rituximab monotherapy was initiated, resulting in clinical improvement, resolution of the constitutional symptoms, and stabilization of ascites.

## 3. Discussion

Diagnosing HHV-8-associated multicentric Castleman disease (MCD) presents a complex challenge, especially in patients with overlapping clinical features, such as underlying immune dysregulation or a history of Kaposi sarcoma (KS) [1,2]. This case underscores the critical need for clinicians to maintain a high index of suspicion when evaluating patients presenting with diffuse lymphadenopathy, systemic symptoms, and polyclonal hypergammaglobulinemia, particularly in the context of HIV infection. The early stages of HHV-8-associated MCD are notoriously difficult to diagnose, because the findings frequently mimic those of more common conditions like lymphoma, autoimmune diseases, or opportunistic infections [1,3]. Despite increasing awareness and the development of new diagnostic standards, HHV-8-associated MCD remains significantly underdiagnosed [1].

### 3.1. Diagnostic Criteria and Clinical Nuances

Fajgenbaum et al. proposed a standardized diagnostic approach for HHV-8-associated MCD, which includes the presence of HHV-8-positive plasmablasts, compatible histopathologic findings, and a constellation of clinical symptoms, such as fever, anemia, hypoalbuminemia, elevated C-reactive protein (CRP), and lymphadenopathy [1]. However, meeting these stringent criteria can be challenging, particularly in early or atypical presentations. Confounding factors, such as recent infections or neurologic syndromes, can further complicate interpretation. In our patient’s case, a recent diagnosis of Guillain–Barré syndrome (GBS) exacerbated the clinical uncertainty. While GBS is not known to trigger MCD, it represents a significant immune disruption that could have obscured or complicated the initial presentation. This highlights the intricate interplay between pre-existing conditions and the manifestation of HHV-8-associated MCD. It is crucial to remember that HHV-8 drives both KS and MCD, but their temporal relationship can vary; they may occur concurrently, or years apart, or in succession [4,7]. The dynamic nature of immune control and viral activity, especially when influenced by antiretroviral therapy or other immunomodulatory treatments, can significantly impact disease progression. Notably, our patient maintained a preserved CD4 count and undetectable HIV viral load throughout, aligning with Hoffmann et al.’s observation that MCD can develop even in patients with well-controlled HIV [2].

### 3.2. The Role of Biopsies in Diagnosis

In at-risk populations, the combination of systemic inflammation, broad-based plasma cell activation, and radiographic lymphadenopathy should immediately raise concerns for HHV-8-associated MCD, as these are typical indicators of the disease [1,2]. Often, core needle biopsies are the initial diagnostic step, due to their relative simplicity and lower procedural risk. While our patient’s core biopsy revealed polytypic plasmacytosis and Castleman-like alterations, it unfortunately lacked sufficient architectural information for a definitive diagnosis. This is a common limitation, as critical diagnostic indicators, such as vascular proliferation, regressed germinal centers, and dispersed HHV-8-positive plasmablasts, can be focal and easily missed without the intact nodal architecture provided by an excisional biopsy [1]. Therefore, when clinical suspicion of HHV-8-associated MCD remains high, as it was in this instance, an excisional biopsy remains the gold standard for diagnosis [1,6].

### 3.3. Impact of Diagnostic Delays and Pathogenesis

The distinction between a core needle and excisional biopsy is critical because delays in performing excisional biopsies frequently prolong the diagnostic timeline, consequently delaying the initiation of essential treatment. In accordance with published consensus criteria, the excisional biopsy for our patient not only confirmed the diagnosis, but also revealed classic MCD histopathologic features, including the characteristic “onion skinning” mantle zones and the presence of LANA-1-positive plasmablasts [1].

The pathogenesis of HHV-8-associated MCD revolves around significant cytokine dysregulation, primarily driven by vIL-6. This viral interleukin-6 mimics human IL-6, relentlessly activating downstream inflammatory pathways [1,4]. This resulting “cytokine storm” is responsible for many of the hallmarks of HHV-8-associated MCD, including fever, ascites, splenomegaly, and laboratory abnormalities, such as anemia, hypoalbuminemia, and polyclonal gammopathy [3,4]. Importantly, the transition of viral latency into a lytic phase can occur independently of the HIV viral load, meaning that immunologic activation is not always proportional to HIV control [3].

### 3.4. Long-Term Considerations and Treatment

The current standard of care treatment for HHV-8-associated MCD is rituximab, an anti-CD20 monoclonal antibody. The work by Gérard et al. and other studies have demonstrated its significant clinical efficacy and tolerability in HIV-positive individuals, leading to the rapid resolution of constitutional symptoms and inflammatory markers [5]. Our patient experienced a marked clinical improvement following the initiation of rituximab, including stabilization of ascites, increased energy levels, and a reduction in lymphadenopathy. While some patients may require additional agents, such as liposomal doxorubicin or antiviral therapy, rituximab monotherapy remains the mainstay for the majority of cases [5]. Oksenhendler et al., in their 20-year review, highlighted the unpredictable course of the disease and the ongoing possibility of relapses, necessitating careful, long-term observation [6]. While improvements in early detection have positively impacted long-term outcomes, timely interventions, the patient’s underlying immune status, and the concurrent presence or absence of KS or other HHV-8-related syndromes remain critical factors influencing survival [6,7].

### 3.5. The Unseen Barriers: Healthcare Accessibility in Rural Settings

The impact of healthcare access on timely diagnosis cannot be overstated. In underserved or rural areas, diagnostic pathways are often fragmented, leading to potential delays in pathology interpretation and specialist referrals. Our patient, residing in rural northern Michigan, exemplifies how individuals with complex diseases may encounter both geographic and systemic barriers to care. These challenges are often compounded by limited health literacy. Furthermore, local laboratories in such regions may lack access to advanced diagnostic techniques like LANA-1 immunohistochemistry, which is crucial for a definitive diagnosis. These limitations can contribute to prolonged clinical decline or the initiation of empirical treatments for alternative conditions, as reflected in Oksenhendler et al.’s observations of variable diagnostic delays across different healthcare environments [6]. Addressing these disparities in healthcare infrastructure and access is vital to improving outcomes for patients with rare and complex conditions like HHV-8-associated MCD.

## 4. Conclusions

This case underscores the importance of a careful workup in patients with unexplained cutaneous, systemic symptoms and lymphoproliferative findings, particularly in those with underlying risk factors like HIV and prior Kaposi sarcoma [2]. The broad and often non-specific nature of the initial symptoms necessitates a high index of suspicion to avoid misdiagnosis or delayed recognition of this aggressive disease. Guillain–Barré syndrome has not been described as a direct trigger for MCD, which added a layer of unique diagnostic uncertainty in this particular case. The patient’s recent immunomodulatory treatment for GBS likely complicated the clinical picture, potentially masking or altering the immune responses pertinent to MCD. These diagnostic challenges are even more pronounced in rural settings, where limited access to specialists, fewer advanced diagnostic facilities, and potentially lower health literacy among patients can further contribute to a significantly delayed diagnosis. When concern for MCD remains high despite inconclusive initial testing, an excisional biopsy should be pursued promptly as the gold standard for a definitive histopathological and immunohistochemical confirmation. Starting rituximab early, immediately upon diagnosis, can lead to a meaningful clinical improvement and, critically, can help stabilize disease progression, profoundly impacting patient outcomes [5,6].

## Data Availability

No new data were created or analyzed in this study. Data sharing is not applicable to this article.

## Abbreviations

The following abbreviations are used in this manuscript:

**Acronym**	**Full Form**
HHV-8	Human Herpesvirus-8
MCD	Multicentric Castleman Disease
KS	Kaposi Sarcoma
HIV	Human Immunodeficiency Virus
AIDS	Acquired Immunodeficiency Syndrome
KSHV	Kaposi Sarcoma-Associated Herpesvirus
PEL	Primary Effusion Lymphoma
LANA-1	Latency-Associated Nuclear Antigen 1
vIL-6	Viral Interleukin-6
IL-6	Interleukin-6
IVIG	Intravenous Immunoglobulin
GBS	Guillain–Barré Syndrome
CD4	Cluster of Differentiation 4 (T-helper cells)
CT	Computed Tomography
H&E	Hematoxylin and Eosin
CD	Cluster of Differentiation (used generally, e.g., CD20)
PCR	Polymerase Chain Reaction
